# Abundance and Seasonal Variations of Snail Intermediate Hosts of Schistosomiasis in the Federal Capital Territory, Abuja, Nigeria

**DOI:** 10.3390/ijerph23030384

**Published:** 2026-03-17

**Authors:** Ifeoma N. Anagbogu, Solomon Monday Jacob, Yoila D. Malann, Ahmed Salihu Dankishiya, Abba Abubakar, Temitope Agbana, Jan-Carel Diehl, Adamu A. Madara

**Affiliations:** 1 Faculty of Science, University of Abuja, Gwagwalada, Abuja 902101, Nigeria; ifechuba@yahoo.co.uk (I.N.A.); malannyd@gmail.com (Y.D.M.); dan-kishiya.ahmed@uniabuja.edu.ng (A.S.D.); adamu.madara@uniabuja.edu.ng (A.A.M.),; 2 Department of Public Health, Federal Ministry of Health, Abuja 900271, Nigeria; 3 Department of Public Health, Federal Capital Territory Administration, Abuja 900247, Nigeria; abubakarabbam@gmail.com; 4 AiDx Medical Bv, 2641 KM Pijnacker, The Netherlands; tope@aidx-medical.com; 5 Sustainable Design Engineering, Delft University of Technology, 2628 CE Delft, The Netherlands

**Keywords:** seasonal variation, snail vector, cercaria shedding, schistosomiasis prevalence

## Abstract

**Highlights:**

**Public health relevance—How does this work relate to a public health issue?**

- Schistosomiasis is a disease of public health importance.
- The disease has been marked for elimination by the WHO on or before 2030.

**Public health significance—Why is this work of significance to public health?**

- The vector and major driver of schistosomiasis is a snail intermediate host.
- The presence of these snails in the studied communities indicates potential risk of infection for humans and other animals.

**Public health implications—What are the key implications or messages for practitioners, policy makers and/or researchers in public health?**

- The study of abundance, seasonal pattern and control of the snail will fast-track the elimination timeline of 2030 set by the WHO.
- Reduced exposure to water bodies with these vectors will contribute towards the reduction or elimination of the disease.

**Abstract:**

One of the strategies for the control and elimination of schistosomiasis is the control of its snail vectors in an endemic area, as is done in other tropical diseases like malaria. However, the strategy currently practiced for the control of the disease in Nigeria is the annual mass administration of preventive chemotherapy (Praziquantel) among school-age children while neglecting the control of its snail intermediate host and other control components. The neglect of malacology and vector control will slow the elimination targets and timeline of 2030 set by the WHO. In this study, we investigated the abundance and seasonal variations in the snail vectors of schistosomiasis and the relationship between the disease among humans and infected snail vectors. A total of 21,282 snails were collected from 13 sites across the six area councils of the Federal Capital Territory (FCT). Of the collected snails, 1451 (6.8%) belong to three species: *Biomphelaria pfeifferi* (0.5%), *Bulinus truncatus* (2.1%) and *Bulinus globosus* (4.2%), which are known to be vectors of *Schistosoma mansoni*, *Schistosoma haematobium* and *Schistosoma bovis*, respectively. These three species were all shedding cercariae both at the time of collection and afterwards, when they were induced to shed cercariae. The association between the reported prevalence of the disease and the percentage of snails shedding cercaria were heterogenous across different communities. While Takushara, with a disease prevalence of 46%, had 60% of the cercaria shedding snails, Kwaita sabo pukafa and Guduji, with disease prevalences of 56% and 26% respectively, had no cercaria shedding snails. Similarly, Dagiri rafin shahu and Gwako 1, with disease prevalences of 60% and 38%, had cercaria shedding snails of less than 1%. Nonetheless, the presence of *Bulinus* and *Biomphelaria* species in these communities indicates a potential risk of infection for humans and other animals who may come in contact with the water. Consequently, integrated multisectoral control and elimination measures that combine malacological monitoring with behavioral, environmental, and historical epidemiological assessments with a deliberate health orientation of the people through sensitization and health education is advocated to reduce exposure to the disease risk factors and contribute towards elimination of the disease.

## 1. Introduction

Schistosomiasis, also known as snail fever, bilharzia and/or Katayama fever, is a parasitic disease caused by blood flukes or parasitic flat worms (trematode worms), of the genus *Schistosoma*. It is one of the neglected tropical diseases (NTDs) earmarked for elimination by the year 2030 by the World Health Organization [1,2,3].

There are two major forms of the disease, intestinal and urogenital, caused by different species of the blood flukes depending on the etiology of the disease. Humans/animals acquire infection from some mollusks or snails that live in freshwater and act as intermediate hosts of these parasites, from which the infective larvae of the parasites escape and pass through the skin of individuals when in contact with the aquatic environment. In humans, the intestinal and urogenital schistosomiasis are caused by *Schistosoma mansoni* and *S. haematobium*, respectively [4], and two genera of freshwater snails, *Biomphalaria* and *Bulinus,* are known to be the intermediate hosts for *Schistosoma mansoni* and *S. haematobium*, respectively [5,6,7]. Both snail genera belong to the Planorbidae family and facilitate the transformation of miracidia into infective cercariae through asexual reproduction. While *Biomphalaria* is primarily linked to intestinal infection, *Bulinus* can also transmit other species like *S. intercalatum* and *S. guineensis* apart from the *S. haematobium*.

Schistosomiasis is prevalent in tropical and subtropical areas [2]; it affects all sexes of different ages, especially women doing domestic chores in infested water, such as washing clothes and plates, or bathing children, especially in poor communities without access to safe drinking water and adequate sanitation where dwellers are constrained from visiting and using the breeding sites of the parasites. People may also get infected by playing and/or swimming or wading through infested water for agricultural and fishing purposes [8,9].

Schistosomiasis remains a major public health concern in Africa, and indeed Nigeria, despite global efforts to eliminate the disease by 2030. According to the WHO [10], the disease is endemic in 78 countries/regions worldwide, with recorded infections in Africa, Asia, the Middle East, and South America. Among the endemic countries/regions, 52 countries experience a moderate-to-high transmission level [11]. The disease is a leading cause of morbidity and mortality in Africa, South America, the Caribbean, the Middle East, and Asia [12], affecting approximately 779 million people globally and resulting in about 280,000 deaths annually [13]. Africa accounts for 93% of the approximately 207 million schistosomiasis cases worldwide, with the highest prevalence in Nigeria, Tanzania, Ghana, Mozambique, and the Democratic Republic of the Congo, totaling up to 78 million cases [10,11,13].

Schistosomiasis is known to be endemic in the FCT, with prevalence established across several communities ranging from as low as 6.1% in Bwari Area Council to as high as 49% in Abuja Municipal Area Council [14]. The complex interplay between environmental factors and human health is increasingly highlighted in the context of infectious diseases, particularly schistosomiasis, which remains a significant public health issue in Nigeria [15]. As the Federal Capital Territory (FCT) grapples with the challenges posed by this disease, a thorough malacological study becomes imperative in understanding the vectors responsible for its transmission. The link between the disease and freshwater environments where host snails proliferate underscores the necessity of investigating these critical organisms. The unique geographical and environmental landscape of the FCT provides an essential backdrop for examining the distribution, ecology, and population dynamics of potential schistosomiasis vectors [14]. Although the prevalence of the disease among humans has been well studied and established across the FCT [14,16,17], other than the study of [18] in a few communities, the abundance and prevalence of the snail intermediate host have not been well studied across the FCT. In this study, we investigated the abundance and seasonal variations in the snail vectors of schistosomiasis and the relationship between the disease among humans and infected snail vectors. To the best of our knowledge, this represents one of the most comprehensive malacological surveys conducted across the FCT.

## 2. Materials and Methods

### 2.1. Study Area

**Figure 1:**
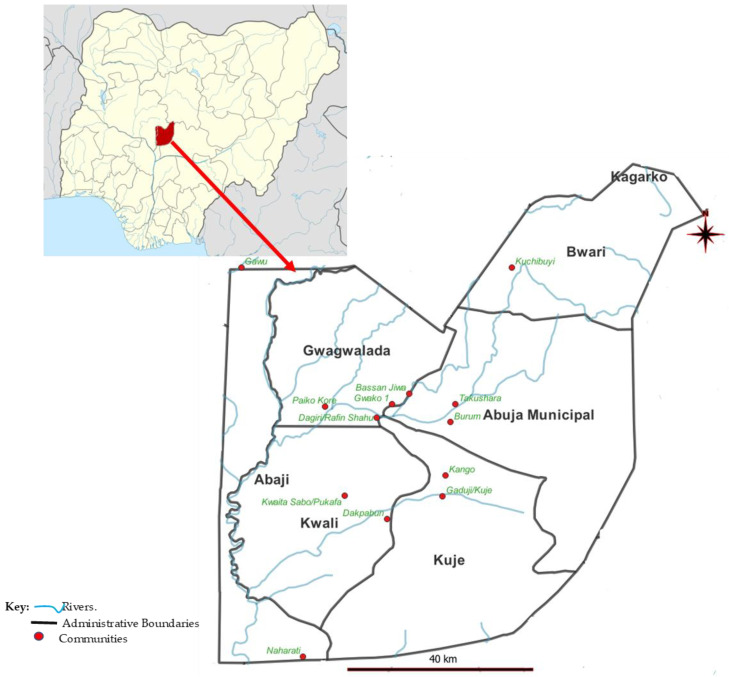
Map of Nigeria showing the study area.

The study was conducted in 13 communities across the 6 area councils (Abaji, Abuja Municipal, Bwari, Gwagwalada, Kuje and Kwali Area councils) of the FCT (Figure 1).

The geographical coordinate of the study area lies between latitude 8.25 and 9.20° N of the equator and longitude 6.45 and 7.39° E of Greenwich meridian. It is situated within the savannah region with moderate climatic conditions. Abuja has a population size of 4,026,000 as of 2024 when projected from the 2006 population census [19]. The primary economic activity in the area is agriculture, which produces crops such as rice, yams, millet, corn, sorghum, and beans. The majority of the population are dairy farmers from the Gwari, Koro, Ganagana, Gwandara, Afo, and Bassa ethnic groups. Hausa and Fulani also live in the territory. While others engage in trading, the city center boast of a sizable number of civil servants who service the seat of governance. Several freshwater habitats intersect the study area, some of which include ponds, streams, dams and tributaries of the Gurara river stretching from Kaduna state. These water bodies form the major source of the water supply to the residents of the study area. During dry seasons, activities increase around these water bodies as people converge to use them for domestic, agricultural and recreational activities, all of which predispose them to schistosomiasis [14].

### 2.2. Study Design and Procedure

#### 2.2.1. Ethical Consideration

Ethical approval for this study was not needed as the study was purely on snail hosts of schistosomiasis and had no human component.

#### 2.2.2. Mapping of Water Bodies Within the Study Area

Maps showing water bodies in the FCT (study area) were prepared using the Arc GIS Version 10.8 and Health mapper Version 4.5 software. The villages and water bodies were validated by personal visits. Thirteen communities known to be endemic for the disease and located close to the mapped water bodies were purposively selected. Geographic coordinates of the selected villages and water bodies were taken using handheld Global Positioning System (GPS) devices, Garmin e-Trex 10 GPS (Olathe, KS, USA), outdoor handheld GPS units, or SMART phones with GPS Camera Apps installed, and documented appropriately. Coordinates of the selected sampling sites on the water bodies were also taken using the same equipment [7,20], as well as photographs of the communities and water bodies.

#### 2.2.3. Selection of Sampling Sites and Collection of Snails

Sampling was conducted in areas about 15–20 m along the banks or perimeter of the selected water bodies and, if they were rivers, from about an area of 5 m^2^ from the water body at each sampling point, especially for *Bulinus* spp. and *Biomphalaria* spp., following [6,7]. The snail collection sites were selected based on their close proximity to human settlements and high level of open defecation and urination. Each of the selected sites were investigated for the presence of freshwater snails in a standardized manner and collections made where they exist. Focal sampling was restricted to places that were commonly used for swimming, bathing, washing and to nearby habitats that were found to harbor snail populations that could aid transmission at the sites. The snails were collected using purpose-built snail scoops and/or small handheld sieves, placed in basins and counted. The snail sampling scoops were standard scoops (2 mm mesh size), and plastic forceps and spoons were used to pick the snails. The scoop was pushed under the vegetation once, lifted up when still under the vegetation and then shaken several times so that the snails were dislodged from the vegetation roots onto the scoop before the scoop was withdrawn [21]. Scooping was performed for 15–45 min from each site, between 6:30 a.m. and 10:00 a.m. for a maximum catch once every month, at the second week of every month for 12 months comprising the rainy season, July to October 2024, and the dry season, November 2024 to June 2025. Samples were collected from several sites along or within the water bodies. The snails attached to vegetation and other substrata, as well as those at the shoreline or banks of the water bodies, were hand-picked wearing gloves [22,23]. The same was the case with the snails that burrowed into the soil. The collected snails were kept in wide-mouthed glass bottles pre-labeled with the community name, and were filled with water and aquatic vegetation from the same area. In some cases, the snails were placed in glass Petri dishes containing wet cotton wool and, where possible, separated accordingly based on the different genera collected. The samples were emptied into a perforated plastic container for transportation to the laboratory at the Department of Biological Sciences, University of Abuja for storage and examination. At the laboratory, snails were sorted, identified and counted following the methods of [24]. The *Schistosoma* snail vectors (*Bulinus* and *Biomphalaria* spp. were further examined for *Schistosoma* spp. infective cercariae, as described by [7,25].

#### 2.2.4. Determination of Snail Abundance and Diversity

The prevalence of infected snail vectors in the rainy and dry seasons was calculated as the abundance and diversity of the different snail species that were collected at the various sampling sites using the Shannon—Weiner diversity index formula [26].

$$H^{\prime }=-\sum_{i=1n}^{s}piln(pi)\;$$

as described by Ref. [26]. Where *H*’ is the index value, *s* is the number of species, *pi* is the proportion of the *i*-th species (*ni*/*N*), and ln is the natural logarithm.

Key Components and Interpretation: *pi* (Proportion): Calculated as

$$\frac{individuals\;of\;species\;i}{total\;individuals\;N}$$

Formula Breakdown: For each species, *pi* ln(*pi*) was computed, values summed and multiplied by −1.

Interpretation: Higher values of *H*′ indicate higher diversity. Values typically range between 1.5 and 3.5, though they can exceed 4.5.

Evenness (*EH*): Measured how similar species abundances were:

$$EH=\frac{H\prime }{{ln(S)}^{\prime }}$$

where *S* is the total number of species.

A higher Shannon index indicates a more diverse, complex, and stable ecosystem, while a value of 0 indicates a community with only one species.

#### 2.2.5. Snail Species Identification

Snails collected from the selected sites were identified using the WHO and other snail identification guides [27,28,29,30]. Other standard protocols for the identification of freshwater snails [31] were also used where necessary and the identification of the snails was mostly based on their morphology and structure. Using the identification keys, most of the snails were identified up to the genus level and, where possible, to the species level, as described in WHO protocols and other studies [27,32]. The common criteria for distinguishing the snail species were the shell shapes, sizes and texture, the nature of the aperture, color and banding pattern of the shells [24,27,29]. A hand lens and dissecting microscope were used in the process.

#### 2.2.6. Screening for Schistosome Infection

Once the morphological identification was completed, the snails were kept in the dark for 48 h preparatory for cercariae shedding induction. At the expiration of the 48 h dark period, the snails were brought out to bright light for cercariae shedding. *Bulinus* spp. and *Biomphalaria* spp. snails were examined for parasitic infection using the shedding method [20]. For this purpose, the snails were placed individually in flat-bottomed glass vials, individual plastic vials, or multi-welled plates containing dechlorinated water, 10 mL of natural spring water [33] with neutral pH or 2 mL of clean and clear water in each of the wells of the multi-well culture plates, and exposed to sunlight for a maximum duration of 4 h, or to artificial light from 60- to 200-W electric bulbs for one to three hours in the absence of sunlight [33]. On the second round of cercaria shedding, the snails were kept at room temperature, preferably in mid-morning, from 10:00 a.m. to 12:00 noon [20,22], as cercariae have a distinct circadian rhythm and the best time to isolate the ones infecting humans is known to be usually mid-morning, about 10:00–12 noon [6,7]. At the end of the shedding period, the wells containing snails were examined under a dissecting microscope. Each well with snails inside was checked for shed cercariae, which have the tendency of making up-and-down movements using their forked coiled tails [23,34]. The live cercariae shed by each snail were transferred to a microscopic slide, covered with a coverslip and carefully observed under a light microscope with ×40 magnification power. Identifications of the cercariae were based on their morphological features using standard identification keys [30,35,36,37,38]. The types and number of cercariae discharged from the snails were properly documented. Cercariae morphologically consistent with human-infective schistosomes were identified based on their distinct morphological features. Non-shedding snails were returned to the ‘aquaria’ for another exposure and examination session the following day before declaring them negative if no cercaria was seen [6]. Based on their morphology, cercariae by *Bulinus* spp. were categorized either as those of *S. haematobium* or those of other trematodes and cercariae from *Biomphalaria* spp. categorized as *S. mansoni* and/or other trematodes. Photographs of the cercariae were taken using the Meubon US Microscope 1 (Houston, TX, USA) 40×–5000× magnification, Digital Imaging, LED Illumination, USB Camera, with mechanical stage, WF10× and WF20× eye pieces and Abbe condenser.

### 2.3. Data Analysis

All raw data collected were entered into an Excel spread sheet for analyses. The Statistical Package for Social Sciences (SPSS) version 25.0 and Epi Info software version 7.2.x were also used for analysis. The prevalence and abundance of infected snails were calculated per collection site, the water body, community, ward and area council. Correlation coefficient (Pearson’s) and the *t*-tests were used to assess the association between the variables, including the seasons and environmental factors, and the snail vector abundance, as well as other covariates (predictor variables), the relationship between the different snail species and the prevalence of schistosomiasis in the study area. The monthly distribution of the snail vectors was analyzed with the Analysis of Variance (ANOVA) for significant difference among the values and also compared with other snail species that were collected at the same site.

## 3. Results

**Figure 2:**
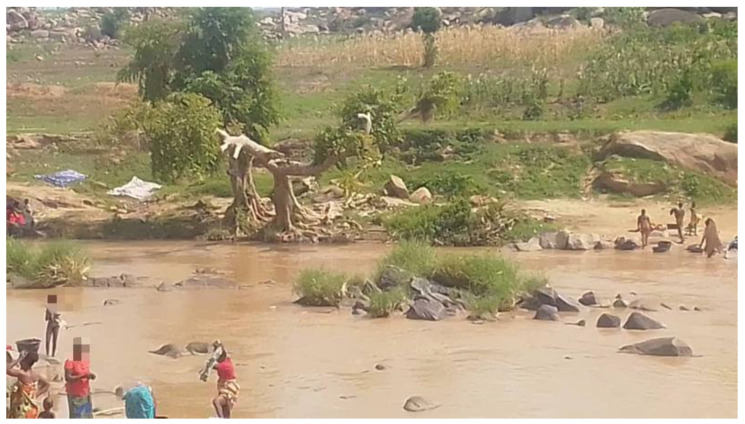
Human water contact activities in River Dagiri of Gwagwalada area council.

The water bodies in the six area councils across the FCT were mapped to identify the distribution of snails (Supplemental Figures S1–S6). Thirteen collection sites were thereafter identified and selected (Figure 1). Farming, cultivation and harvesting of rice were ongoing in some of these water bodies during the period of study. Human activities were also seen around some of the water bodies (Figure 2).

### 3.1. Abundance of Snails by Species

**Figure 3:**
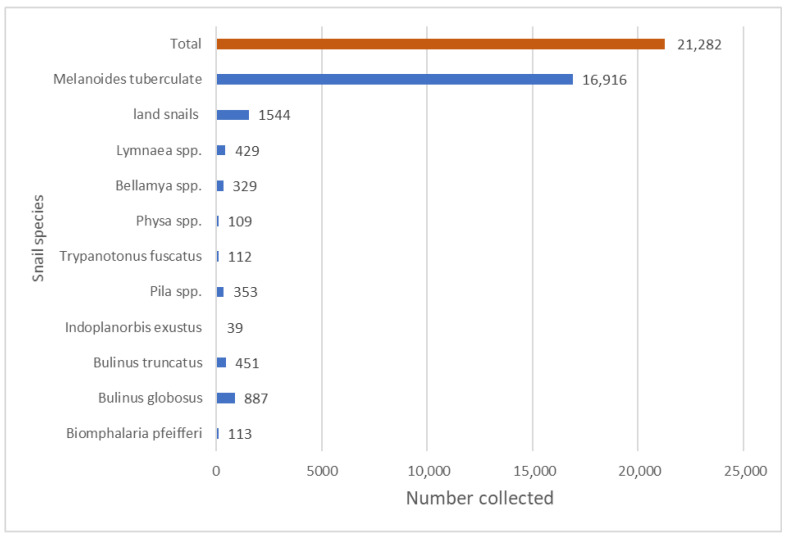
Abundance of snail species collected.

A total of 21,282 snails were collected and identified from the sampling sites. The snails were in the Phylum Mollusca, Class Gastropoda and Sub Class Pulmonata. They belong to the Families of: Bulinidae Thiaridae, Lymnaeidae, Planorbidae, Viviparidae, Physidae, Potamididae, Ampullaridae and Achatinidae. The species collected include *Bulinus globosus*, *Bulinus truncatus*, *Biomphalaria pfeifferi*, *Indoplanorbis exustus*, *Melanoides tuberculata*, *Bellamya* spp., *Pila* spp., *Lymaea* spp., *Physa* spp. and *Tympanotonus fuscatus*. Different species of land snails were also collected from the surveyed sites. *Melanoides* spp. were the most abundant, with 16,916 (79.5%) collected, and *Indoplanorbis exustus* the least, with 39 (0.2%) (Figure 3).

**Figure 4:**
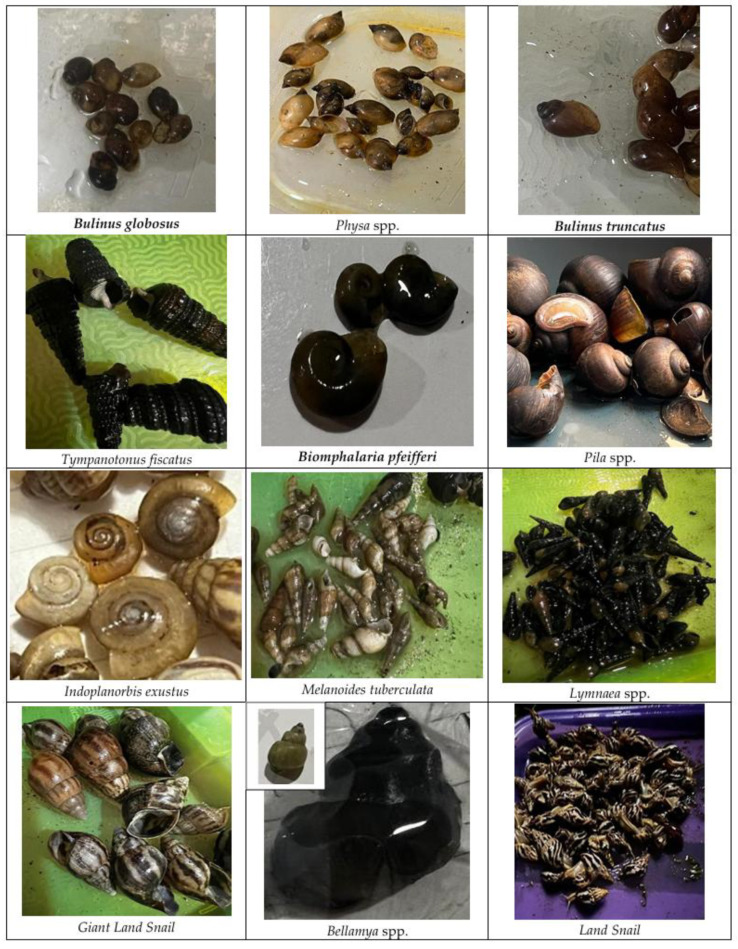
Snails collected between July 2024 and June 2025.

Figure 4 shows the photographs of snails collected between July 2024 and June 2025. Among the snails collected, only the *Bulinus* and *Biomphalaria* spp. are known to be intermediate hosts for schistosomiasis.

### 3.2. Snail Abundance by Months

The highest abundance of snails was recorded at the peak of the raining season, the month of August 2024, with a total of 3175 (14.9%) snails collected while the least, 322 (1.5%), was collected in the month of January 2025 (Table 1).

### 3.3. Distribution of Snails’ Species by Communities

At least one of the schistosomiasis intermediate hosts or vectors, *Bulinus* and *Biomphalaria* spp., were collected in all the communities except Kuje and Pukafa. *Melanoides tuberculata* had the highest occurrence with 16,916 and had the highest occurrence across all the communities, while *Indoplanorbis exustus* had the least occurrence (Table 2). The highest number of snails, 6460 (30.4%), was collected from Gwarko 1, while the least number of snails, 218 (1.0%), were collected from Gawu village in Abaji Area Council.

### 3.4. Seasonal Variation in Snail Intermediate Host of Schistosoma Species

Overall, the snails were more abundant during the wet season (11,723) compared to the 9559 collected during the dry season. Takushara recorded the highest abundance with 61.19% during the wet and 54.98% in the dry season. Similarly, Burum recorded a high abundance of 39.62% during the wet and 12.99% during the dry season. On the other hand, Bassan Jiwa and Kango, with zero abundance during the wet season, recorded a dry season abundance of 37.8% and 18.56%, respectively. All snails collected during the wet and dry seasons were subjected to cercaria shedding. However, during the wet season, significant cercaria shedding was only observed in two communities, Takushara (61%) and Burum (39.6%), in contrast with the dry season, where shedding of cercaria was observed in most of the communities (Table 3).

### 3.5. Relationship Between Disease Endemicity and Snail Shedding Cercaria

**Figure 5:**
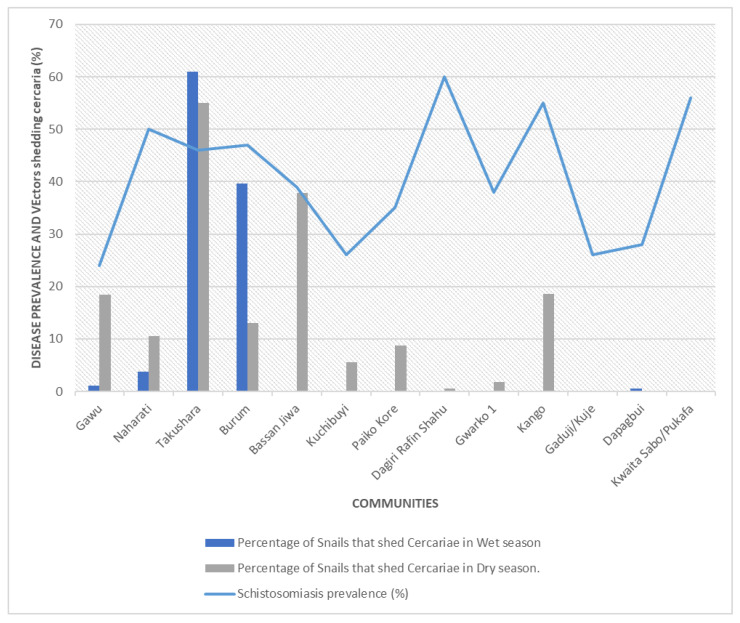
Relationship between disease prevalence and percentage of vectors shedding Cercaria.

There was an association between the prevalence of the disease and the percentage of snails shedding cercaria in Takushara, Bassan Jiwa, Burum and Gawu communities. However, the association between the disease and cercaria shedding in other communities was not significant. Based on the disease endemicity as reported by [14,15], while Takushara, with a disease prevalence of 46%, had 60% of snail shed cercaria, Kwaita sabo pukafa and Guduji, with disease prevalences of 56% and 26% respectively, had no cercaria shedding snails from these communities. Similarly, Dagiri rafin shahu and Gwako 1, with disease prevalences of 60% and 38%s had cercaria shedding snails of less than 1% (Figure 5).

## 4. Discussion

Schistosomiasis remains one of the world’s most prevalent diseases of public health importance. Despite more than a century of control efforts and the introduction of highly effective anti-schistosomal drugs, the eradication of the disease is still far from actualization. The disease is one of the neglected tropical diseases targeted for elimination by 2030 according to the WHO roadmap 2030 [1]. Consequently, each endemic country is working at meeting this target by reviewing its strategies for elimination. One such strategy is control of the vectors, especially the *Bulinus* and *Biomphelaria* species that have been implicated in the transmission of schistosomiasis.

The identification and verification of water bodies for schistosomiasis vectors within the six area councils of the Federal Capital Territory (FCT) was intended to facilitate targeted interventions by identifying water bodies that harbor the vectors and allows health authorities to implement localized control measures, such as molluscicide or environmental management to reduce transmission [38]. It will also facilitate monitoring of high-risk areas and help in directing resources efficiently, thereby improving the effectiveness of ongoing surveillance and early detection of outbreaks [1]. Consequently, knowledge of specific water bodies linked to schistosomiasis transmission will promote community awareness and behavioral changes, such as avoiding contact with such contaminated water sources [39]. This identification will also inform environmental modifications or infrastructural improvements to reduce breeding sites and support evidence-based policymaking for integrated schistosomiasis control strategies at local and national levels [40]. During the course of this study—July 2024 to June 2025—a total of 21,282 snail samples were collected, out of which 1451 (6.8%) belong to three species *Biomphelaria pfeifferi* (113), *Bulinus truncatus* (451) and *Bulinus globosus* (887), that are known to be vectors of schistosomiasis. These three species were all shedding cercariae both at the time of collection and afterwards when they were induced to shed cercariae. The presence and shedding of cercaria by the *Bulinus* and *Biomphelaria* species in the studied communities indicates potential risk of infection for humans and other animals who may come in contact with the water. This agrees with the findings of [41] in Borno State, Nigeria, where infection with schistosomiasis was linked to the presence of cercariae shedding *Bulinus* and *Bionphelaria* spp. Although the presence of these snail vectors was established in all the study villages except in Kuje and Pukafa, in some communities, there were no infected snails as they did not shed cercaria both in the wet and dry seasons. Nonetheless, a deliberate health orientation of the people through sensitization and health education activities, the provision of safe and adequate water sources and other WASH amenities to reduce exposure to the disease risk factors will contribute towards the reduction or elimination of the disease in the communities.

The collection of 900 snail vectors of schistosomiasis in the dry season as against the 551 in the wet season supports the seasonal variation in the human *Schistosoma* spp. vectors. These findings align with the work of [42] in the Niger River Valley, where it was shown that seasonality in abundance was statistically significant in all species, with greater numbers associated with dry season months in the first half of the year, but are contrary to the findings of [43] in Senegal, where snail abundance was lowest in the early dry season and peaked during the rainy season. The findings in this study may have been influenced by the fact that, during dry seasons, many temporary water bodies shrink or dry up; this can both reduce habitat and concentrate snails where water remains. In perennial habitats, the pattern may be different [44]. Ephemeral habitats may exist during the rainy season but may be disturbed or flushed out and snail survival can be low if flows are strong [42]. In addition, aquatic vegetation provides habitat and shelters, and periphyton (algae-biofilms) are food. These tend to increase after rains, but may also be more stable in the dry season in some settings [43]. These findings imply that factors such as historical exposure patterns, seasonal water contact behavior, environmental variability, and focal snail distribution may influence transmission dynamics beyond current snail infection rates. Consequently, integrated multisectoral control and elimination measures that combine malacological monitoring with behavioral, environmental, and historical epidemiological assessments are advocated. The main limitation of this study is the inability to subject the cercaria to molecular confirmation for all cercariae types, but it relied solely on microscopic identification.

## 5. Conclusions

The association between the reported prevalence of the disease and the percentage of snails shedding cercaria were heterogenous across different communities. Nonetheless, the presence of *Bulinus* and *Biomphelaria* species in these communities indicates potential risk of infection for humans and other animals who may come in contact with the water. Findings from this study demonstrate that, in the FCT, schistosoma transmission remains a challenge. These data provide an actionable map—ecological and programmatic—for shifting from routine control to true elimination-aligned operations in Abuja. Since environmental signals differ by season, an integrated, multi-season surveillance that pairs malacology with behavioral and mobility data targeting snail control, and concurrent WASH, will drastically reduce hotspots. Government and non-governmental organizations are encouraged to provide alternate sources of water-like boreholes or pipe-borne water, to reduce frequent water contact activities and implement a behavioral change campaign across the communities to control and eliminate the disease. We recommend cautions against interpreting single-season or single-parameter snail metrics as direct proxies for human disease risk in heterogeneous, highly seasonal systems.

## Figures and Tables

**Table 1:** Monthly variation in snails in the villages; July 2024–June 2025.

Months													
Villages/Communities	Jul 24 (%)	Aug 24 (%)	Sep 24 (%)	Oct 24 (%)	Nov 24 (%)	Dec 24 (%)	Jan 25 (%)	Feb 25 (%)	Mar 25 (%)	Apr 25 (%)	May 25 (%)	June 25 (%)	Total Snails by Community
Gawu	2	117	26	35	32	6	0	0	0	0	0	0	218
Naharati	73	10	32	26	65	45	0	0	13	1	21	0	286
Takushara	158	118	171	124	137	146	54	25	30	30	0	68	1061
Burum	20	149	0	147	11	195	241	164	14	14	0	50	1005
Bassan Jiwa	0	0	0	0	65	11	12	130	59	59	153	570	1059
Kuchibuyi	38	66	24	8	67	65	15	22	0	276	46	41	668
Paiko Kore	379	137	45	0	20	140	0	272	600	95	200	500	2388
Dagiri Rafin Shahu	82	227	14	23	0	109	0	178	689	546	73	350	2291
Gwarko 1	863	883	566	17	354	691	0	698	620	356	392	1020	6460
Kango	0	7	36	0	7	6	0	546	349	482	55	179	1667
Gaduji/Kuje	24	1422	516	425	5	8	0	8	0	0	6	40	2454
Dapagbui	71	39	503	17	47	108	0	48	84	84	88	154	1243
Kwaita Sabo/Pukafa	42	0	0	35	185	220	0	0	0	0	0	0	482
	1752 (8.2)	3175 (14.9)	1933 (9.1)	857 (4.0)	995 (4.7)	1750 (8.2)	322 (1.5)	2091 (9.8)	2458 (11.5)	1943 (9.1)	1034 (4.9)	2972 (14)	21,282

**Table 2:** Distribution of snail species by communities.

Villages/Communities	BF	BG	BT	INE	MT	LDS	PS	TF	PHS	BS	LS	Total Snails by Community
Gawu	2	0	7	0	171	13	0	0	0	25	0	218
Naharati	5	2	12	0	42	125	55	3	29	13	0	286
Takushara	66	507	50	0	112	319	7	0	0	0	0	1061
Burum	25	131	72	6	458	131	13	0	31	28	110	1005
Bassan Jiwa	0	71	56	0	866	58	0	8	0	0	0	1059
Kuchibuyi	13	0	12	15	485	68	0	13	0	6	56	668
Paiko Kore	0	0	99	0	2006	86	81	0	0	0	116	2388
Dagiri Rafin Shahu	0	10	0	18	1908	275	10	22	0	9	39	2291
Gwarko 1	0	41	7	0	6041	163	81	32	0	0	95	6460
Kango	0	122	136	0	1338	28	39	4	0	0	0	1667
Gaduji/Kuje	0	0	0	0	2303	54	67	30	0	0	0	2454
Dapagbui	2	3	0	0	839	173	0	0	49	164	13	1243
Kwaita Sabo/Pukafa	0	0	0	0	347	51	0	0	0	84	0	482
Total	113	887	451	39	16,916	1544	353	112	109	329	429	21,282

Key: BF—*Biomphalaria pfeifferi*, BG—*Bulinus globosus*, BT—*Bulinus truncatus*, INE—*Indoplanorbis exustus*, MT—*Melanoides tuberculate*, LDS—land snails, PS—*Pila* spp., TF—*Trypanotonus fuscatus*, PHS—*Physa* spp., BS—*Bellamya* spp., LS—*Lymnaea* spp.

**Table 3:** Abundance and cercaria shedding snails during wet and dry seasons by communities.

Village/Communities	NSW	NSD	NSCW (%)	NSCD (%)	Wet Abundance (%)	Dry Abundance (%)
Gawu	180	38	2 (1.1)	7 (18.4)	1.11	18.42
Naharati	162	124	6 (3.7)	13 (10.5)	3.7	10.48
Takushara	639	422	391 (61)	232 (55)	61.19	54.98
Burum	366	639	145 (39.6)	83 (13)	39.62	12.99
Bassan Jiwa	723	336	0	127 (37.8)	0	37.8
Kuchibuyi	223	445	0	25 (5.6)	0	5.62
Paiko Kore	1261	1127	1 (0.1)	98 (8.7)	0.08	8.7
Dagiri Rafin Shahu	769	1522	1 (0.1)	9 (0.6)	0.13	0.59
Gwarko 1	3741	2719	0	48 (1.8)	0	1.77
Kango	277	1390	0	258 (18.6)	0	18.56
Gaduji/Kuje	2433	21	0	0	0	0
Dapagbui	872	371	5 (0.6)	0	0.57	0
Kwaita Sabo/Pukafa	77	405	0	0	0	0
	11,723	9559	551	900		

Key: NSW—number of snails in wet season, NSD—number of snails in dry season, NSCW—number of snails that shed cercariae in wet season, NSCD—number of snails that shed cercariae in dry season.

## Data Availability

The original contributions presented in this study are included in the article. Further inquiries can be directed to the corresponding authors.

## Supplementary Materials

The following supporting information can be downloaded at: https://www.mdpi.com/article/10.3390/ijerph23030384/s1, Figure S1: Verified water bodies in Abaji Area Council; Figure S2: Verified water bodies in Abuja Municipal Area Council; Figure S3: Verified water bodies in Bwari Area Council; Figure S4: Verified water bodies in Gwagwalada Area Council; Figure S5: Verified water bodies in Kuje Area Council; Figure S6: Verified water bodies in Kwali Area Council.
