# Supplementary material for: Abundance and Seasonal Variations of Snail Intermediate Hosts of Schistosomiasis in the Federal Capital Territory, Abuja, Nigeria
Source: Int J Environ Res Public Health. 2026 Mar 17;23(3):384. doi: 10.3390/ijerph23030384 (PMC13026216; doi:10.3390/ijerph23030384)
Supplement: Supplementary file 1 [file ijerph-23-00384-s001.zip › ijerph-4087182-supplementary.pdf]

Supplementary figures

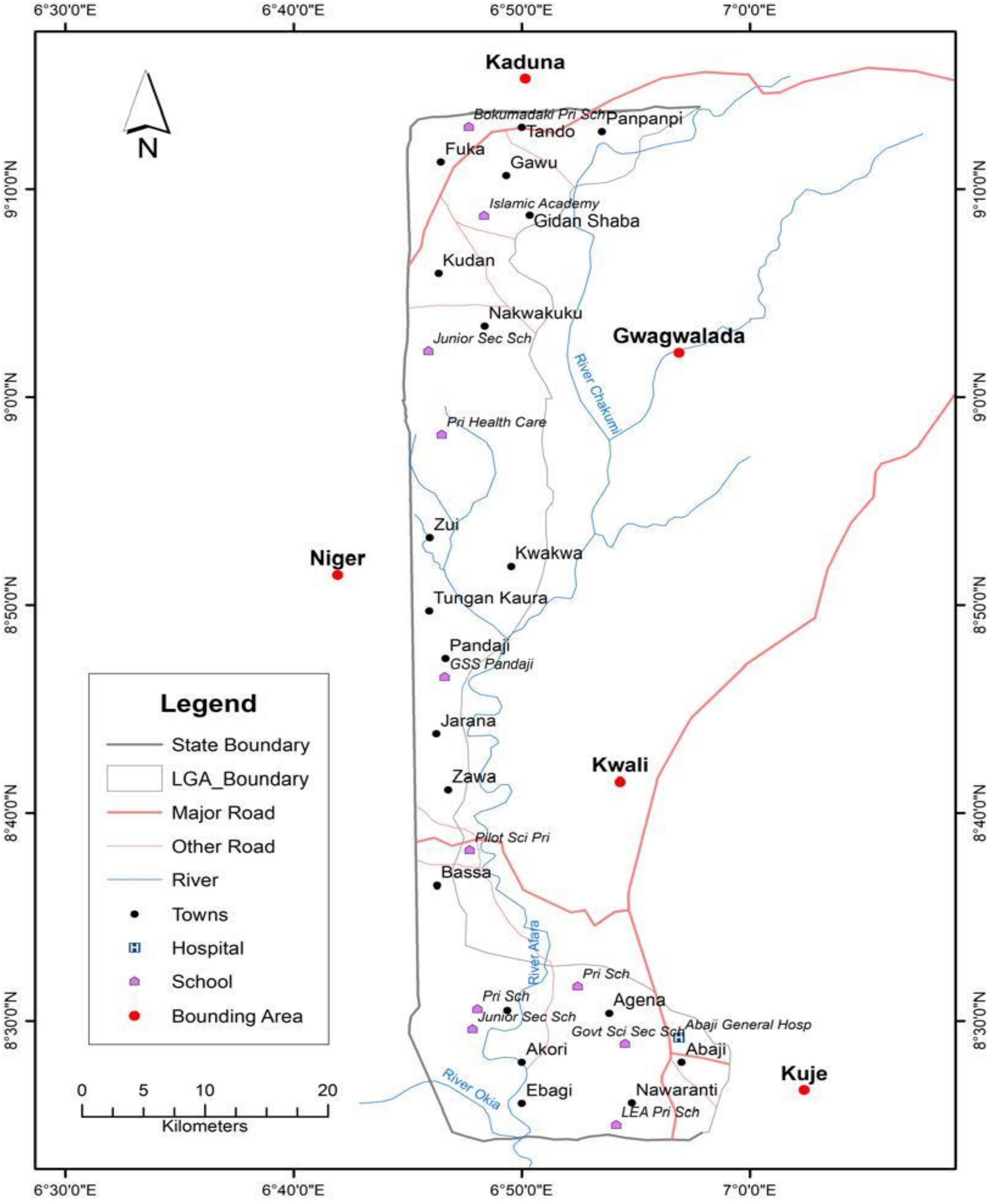

Surplemental Figure S1. Verified water bodies in Abaji Area Council

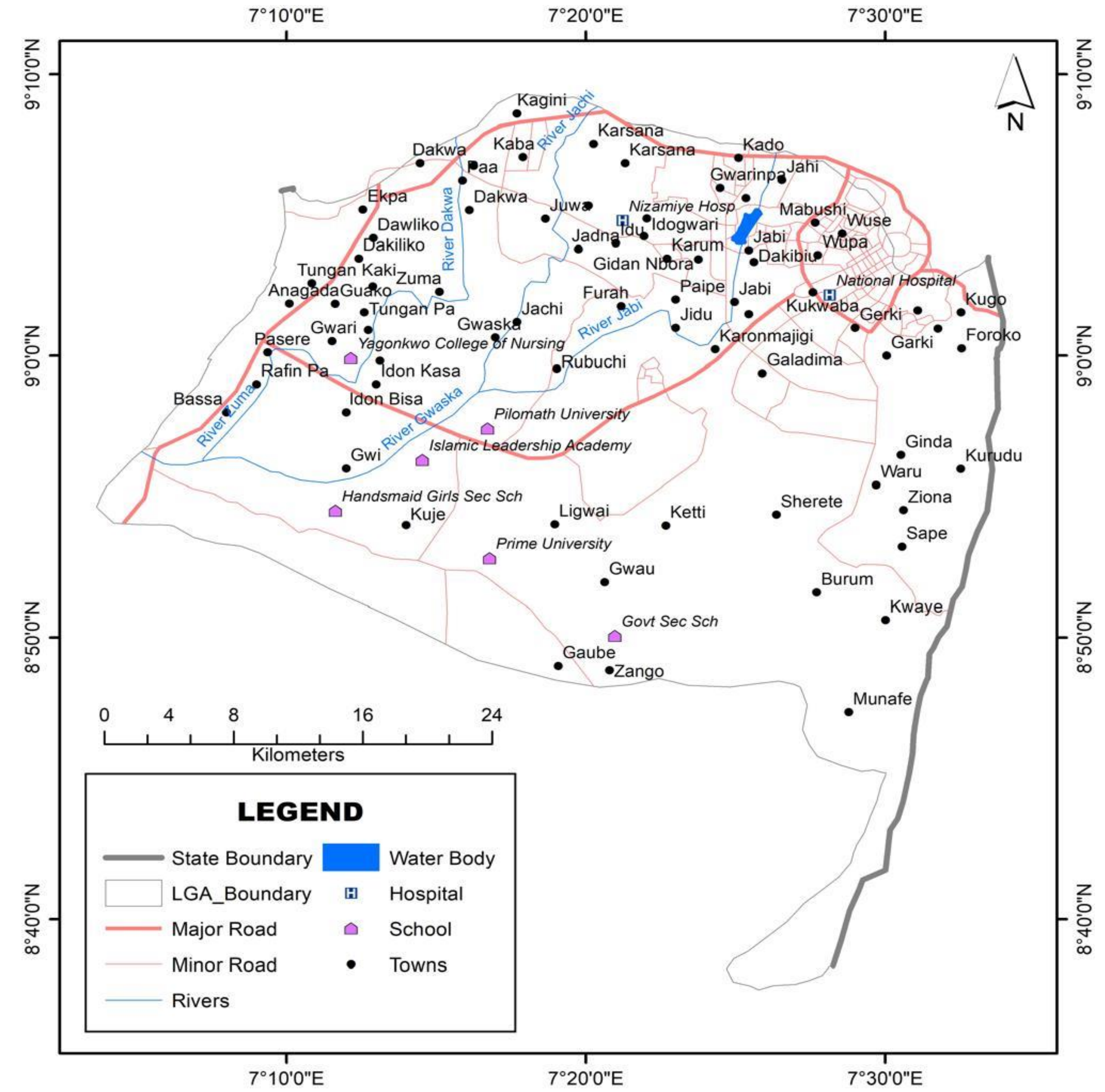

Surplemental Figure S2. Verified water bodies in Abuja Municipal Area Council

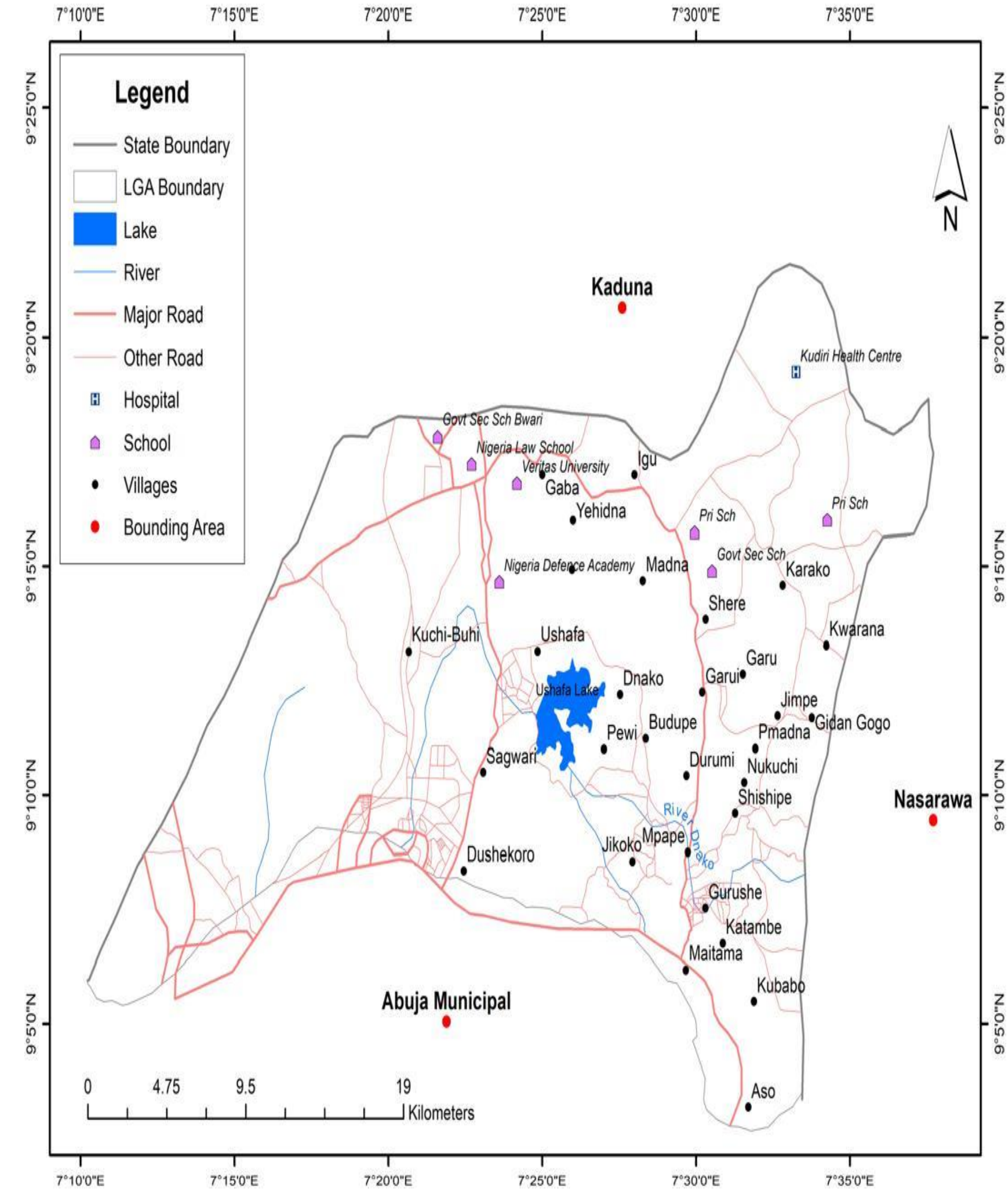

Surplemental Figure S3. Verified water bodies in Bwari Area Council

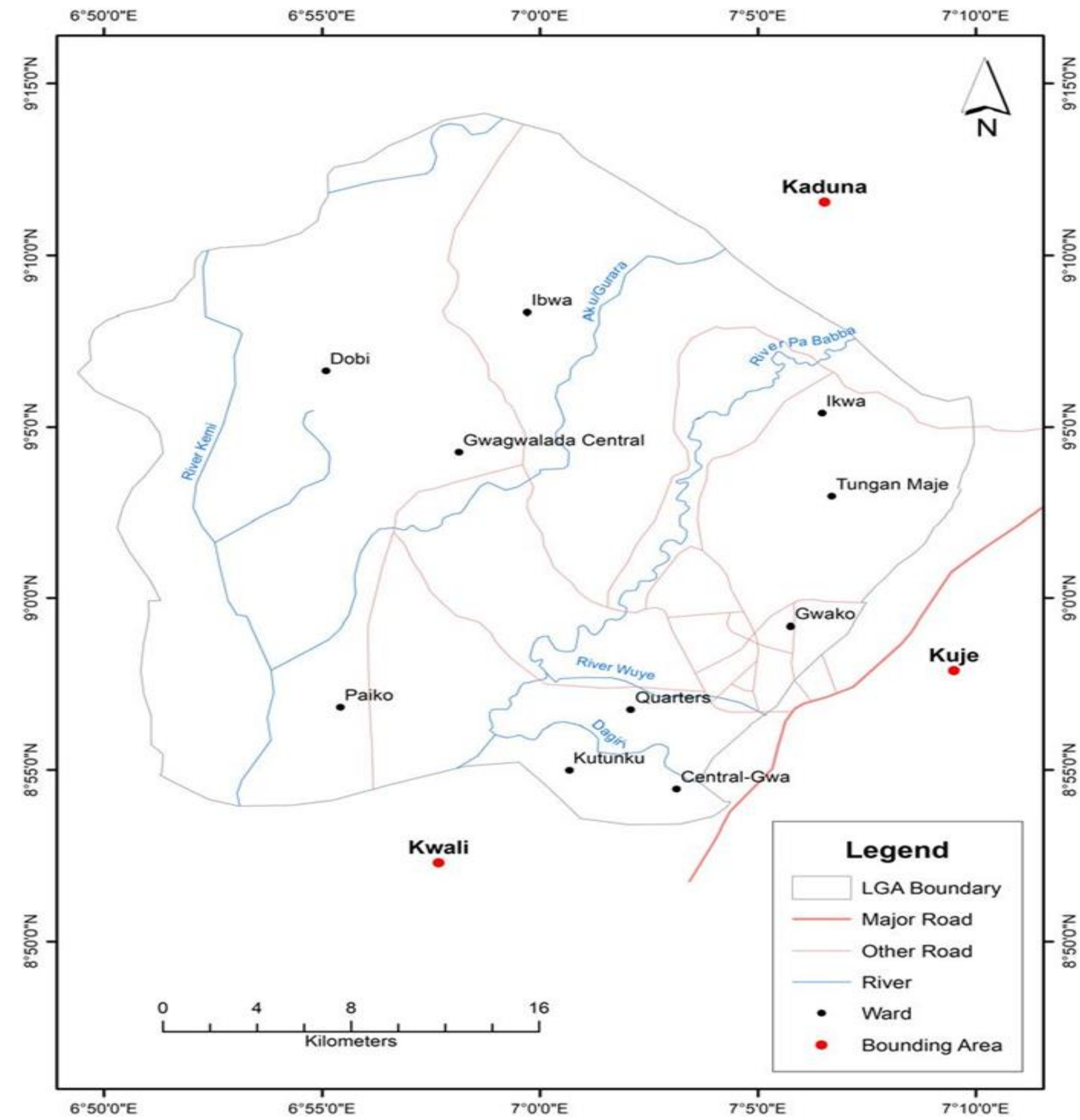

Surplemental Figure S4. Verified water bodies in Gwagwalada Area Council

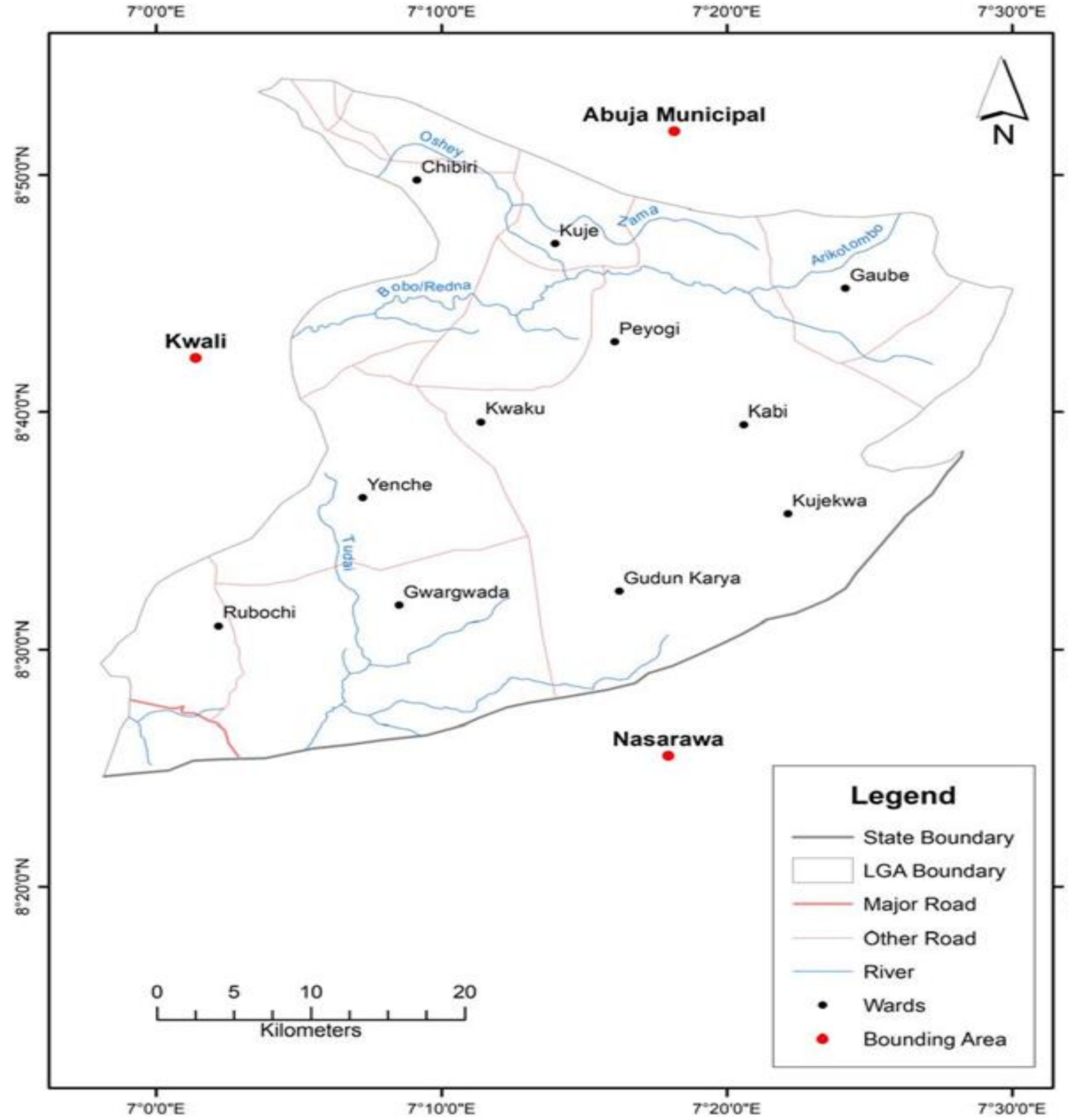

Surplemental Figure S5: Verified water bodies in Kuje Area Council

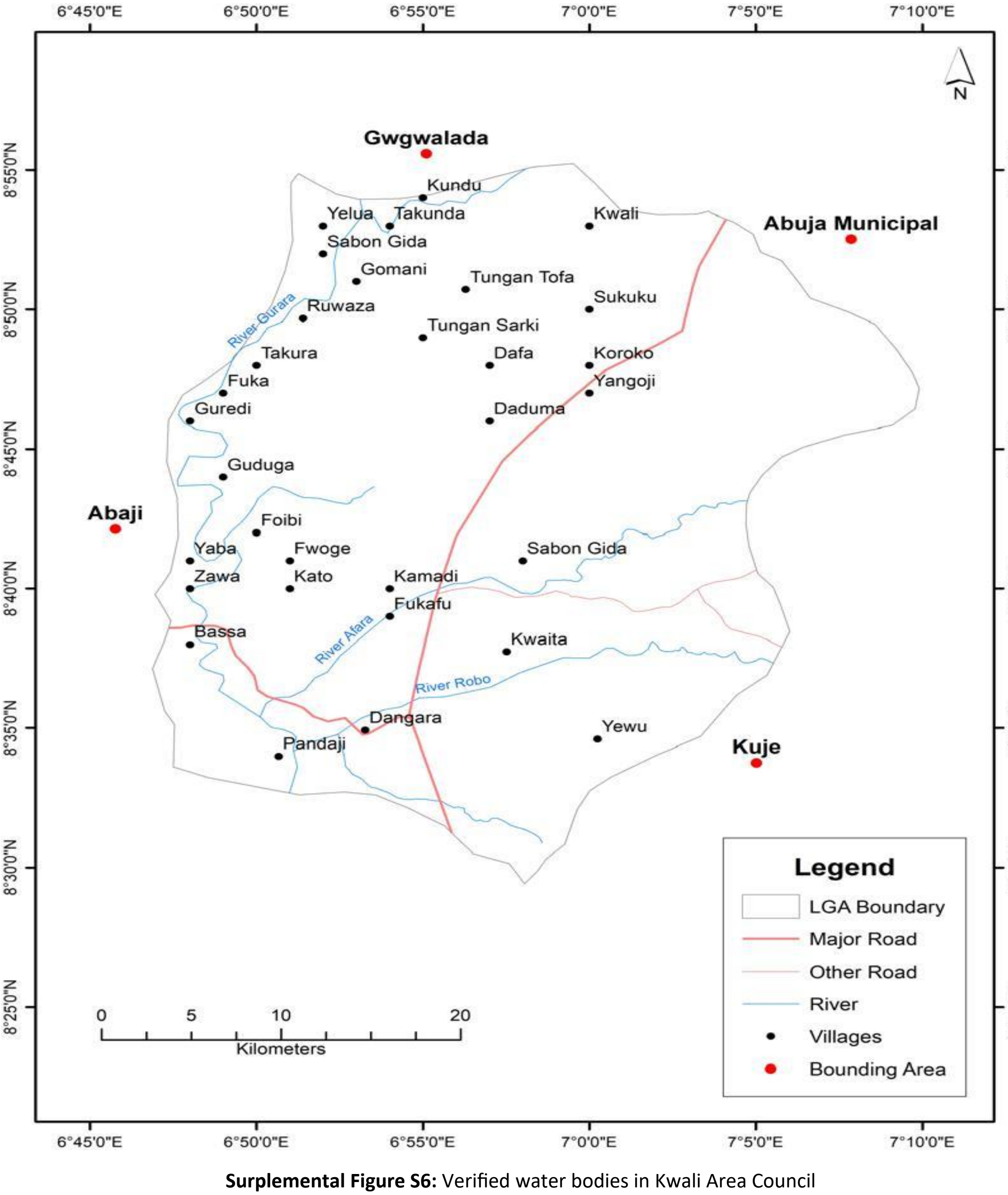

Surplemental Figure S6: Verified water bodies in Kwali Area Council
